# Phase I Randomized Study of a Tetravalent Dengue Purified Inactivated Vaccine in Healthy Adults from Puerto Rico

**DOI:** 10.4269/ajtmh.17-0627

**Published:** 2018-03-05

**Authors:** Clemente Diaz, Leyi Lin, Luis J. Martinez, Kenneth H. Eckels, Maribel Campos, Richard G. Jarman, Rafael De La Barrera, Edith Lepine, Jean-François Toussaint, Irma Febo, Bruce L. Innis, Stephen J. Thomas, Alexander C. Schmidt

**Affiliations:** 1 University of Puerto Rico School of Medicine, San Juan, Puerto Rico;; 2 Walter Reed Army Institute of Research, Silver Spring, Maryland;; 3 GlaxoSmithKline, Rockville, Maryland;; 4 GlaxoSmithKline, Rixensart, Belgium

## Abstract

The safety and immunogenicity of four adjuvanted formulations of an investigational tetravalent dengue purified inactivated vaccine (DPIV) were evaluated in a predominantly dengue-primed population in Puerto Rico. In this placebo-controlled, randomized, observer-blind, phase I trial, 100 healthy adults were randomized 1:1:1:1:1 to receive DPIV at Day (D)0 and D28 (1 μg per dengue virus [DENV] type 1–4 adjuvanted with either alum, AS01_E_ or AS03_B_, or 4 μg per DENV type adjuvanted with alum) or saline placebo. Functional antibody responses were assessed using a microneutralization assay at D56, Month (M)7, and M13. All DPIV formulations were well tolerated and no safety signals were identified through M13. The M13 according-to-protocol (ATP) immunogenicity cohort included 83 participants. The ATP analysis of immunogenicity was performed only on the 78 subjects seropositive for ≥ 1 DENV type at baseline: 69 tetravalent, three trivalent, two bivalent, and four monovalent. In all DPIV groups, geometric mean antibody titers (GMTs) increased from D0 to D56 and waned modestly through M13, while remaining well above prevaccination levels. The 4 μg + alum and the AS01_E_- and AS03_B_-adjuvanted formulations were highly immunogenic, with M13-neutralizing antibody GMTs against all four DENV types above 1,000. M13/D0 GMT ratios were highest in the 1 μg + AS03_B_ group (ranging 3.2–3.7 depending on the DENV type). These results encourage continued clinical development of DPIV (ClinicalTrials.gov: NCT01702857).

## INTRODUCTION

Dengue is a mosquito-borne viral disease found in tropical and subtropical climates worldwide. Dengue is caused by any of the four single stranded, positive-sense enveloped RNA viruses (dengue virus [DENV]-1, -2, -3, and -4) from the genus *Flavivirus*. Global dengue incidence has increased 30-fold in the last 50 years,^1^ with 390 million dengue infections estimated to occur every year, of which 96 million are clinically apparent.^2^ Dengue fever is endemic in Puerto Rico and transmission intensity varies geographically and by season.^3,4^ A recent vaccine study in Puerto Rico found that the vast majority of young adults were seropositive for dengue and 89% of 21–50-year-olds were tetravalent seropositive.^5^ In 2010, Puerto Rico experienced the largest dengue outbreak in its history, with more than 26,000 suspected cases reported.^3^ Although endemic dengue cases are seen every year, cycles of epidemic outbreaks have been reported with increasing intensity during the last decades.

A live-attenuated dengue vaccine, based on yellow fever/dengue chimeras, has recently been licensed in a number of countries for use in adults and in children aged 9 years and older, and the World Health Organization (WHO) has issued recommendations on potential use scenarios in areas with high seroprevalence.^6^ Two additional live-attenuated dengue vaccines are in phase III development (ClinicalTrials.gov: NCT02747927 and NCT02406729).

The Walter Reed Army Institute of Research (WRAIR), Fiocruz, and GlaxoSmithKline (GSK) are presently collaborating in the development of a tetravalent dengue purified inactivated vaccine (DPIV) candidate. Previously, WRAIR and GSK had collaborated for more than a decade to evaluate a live-attenuated vaccine (LAV) candidate that had a promising safety and immunogenicity profile in phase I and II trials involving infants, children, and adults.^5,7–13^ To overcome some of the perceived shortcomings of the LAV approach (prior dengue exposure changes vaccine virus replication, difficult to achieve a balanced efficacy against infection or disease with each DENV type postvaccination, potential for more severe disease in dengue-naive vaccinees),^14^ we decided to focus our collaborative efforts on an adjuvanted tetravalent DPIV candidate, aiming for a short immunization schedule, a balanced humoral immune response to the four DENV serotypes, a rapid onset of protection, and to maintain the ability to boost responses if needed. Several adjuvanted DPIV formulations were highly immunogenic in nonhuman primates (NHPs), even at relatively low antigen dose levels.^15^ Two doses of DPIV administered 4 weeks apart protected NHPs from viremia after a challenge with low-passage clinical isolates of DENV-1 and DENV-2 40 and 32 weeks postdose 2, respectively.^15^

As a part of the clinical development of a tetravalent DPIV candidate, WRAIR and GSK initiated two phase I clinical trials in endemic and nonendemic regions using four different DPIV vaccine formulations adjuvanted with either aluminum hydroxide (alum) or GSK’s adjuvant systems AS01_E_ or AS03_B_ administered intramuscularly (IM) as a 2-dose schedule (Day [D]0 and D28). The first trial (NCT01666652), conducted in predominantly flavivirus-naive healthy adults, showed that the adjuvanted tetravalent DPIV candidates had an acceptable safety profile and induced high titer balanced neutralizing antibody responses at 1 month postdose 2 that waned considerably by Month (M)7.^16^ Another clinical study showed that a monovalent DPIV candidate (DENV-1), adjuvanted with alum, was generally well tolerated and had an acceptable immunogenicity profile in a small number of healthy flavivirus-naive adults.^17^

The phase I trial reported here was conducted in predominantly dengue-primed healthy adults in Puerto Rico. Although study subjects will be followed for safety for a total of 3 years after vaccination, we present here the safety, reactogenicity, and humoral immunogenicity data of the four DPIV formulations generated during the first 13 months of the study.

## MATERIALS AND METHODS

### Study design.

This study was a phase I, randomized, placebo-controlled, and observer-blind primary vaccination trial designed to evaluate the safety and immunogenicity of four DPIV vaccine formulations, administered as two doses 4 weeks apart, conducted in Puerto Rico in a predominantly dengue-primed population (Clinicaltrials.gov: NCT01702857).

The primary objectives were as follows: 1) to evaluate the safety and reactogenicity of four DPIV formulations from D0 through 28 days after the second vaccine dose (D56) and 2) to evaluate the humoral immunogenicity to each DENV type at D56. The secondary objectives were as follows: 1) to evaluate the safety of the four DPIV formulations from D0 through 12 months after the second vaccine dose (M13) and 2) to evaluate the humoral immunogenicity to each DENV type up to M13.

The study was conducted at the University of Puerto Rico Medical Sciences Campus, Puerto Rico Clinical and Translational Research Consortium in accordance with the Declaration of Helsinki, International Conference on Harmonization, Good Clinical Practice, Belmont Principles, and other applicable regulatory and Department of Defense requirements. The protocol and associated documents were reviewed and approved by the U.S. Army Human Subjects Research Review Board, Office of the Surgeon General, the U.S. Army Medical Materiel Development Activity (USAMMDA), and GSK. The Western Institutional Review Board, Puyallup, WA, reviewed the protocol and supporting documents on behalf of the University of Puerto Rico. Internal audits by separate teams from the U.S. Army and GSK were also conducted. All participants provided written informed consent before study entry.

### Role of the sponsor and development partners.

This study was designed and the protocol developed by the WRAIR and GSK. The study was co-funded by the U.S. Army Medical Research and Materiel Command, Military Infectious Diseases Research Program, and by GSK Biologicals SA. The USAMMDA, as the Office of the Surgeon General’s (sponsor) representative, monitored and reported on subject safety. Data management and statistical analysis were performed at GSK, according to prespecified and mutually approved plans. A blinded safety review team and an unblinded safety review committee reviewed safety data at scheduled intervals. All the authors reviewed the article and vouch for its accuracy and completeness.

### Vaccines.

The DPIV vaccine used in this study consisted of adjuvanted, purified, and inactivated tetravalent virus strains (DENV types 1–4) produced in Vero cells. The preparation of the DPIV antigens has been described previously.^18,19^ Briefly, DPIV consists of a tetravalent formulation of the following nonattenuated DENV strains: West Pac 74 (DENV-1), S16803 (DENV-2), CH53489 (DENV-3), and TVP360 (DENV-4), propagated in Vero cells, purified, and inactivated with formalin.

Adjuvants used were alum (Alhydrogel 2%; Brenntag Biosector, Frederikssund, Denmark; 10.38 mg of Al^3+^/mL; after dilution, each 0.5 mL vaccine dose contains 500 μg of Al^3+^), and the adjuvant systems AS01_E_ and AS03_B_ supplied by GSK, Rixensart, Belgium. AS01_E_ is an adjuvant system containing 3-O-desacyl-4′-monophosphoryl lipid A (MPL; GSK), QS-21 (*Quillaja saponaria* Molina, fraction 21) (Licensed by GSK from Antigenics LLC, a wholly owned subsidiary of Agenus, Inc., a Delaware, United States corporation), and liposome (25 μg MPL and 25 μg QS-21). AS03_B_ is an adjuvant system containing α-tocopherol and squalene in an o/w emulsion (5.93 mg α-tocopherol).

Four different formulations of the DPIV vaccine were used: 1 μg/serotype/dose adjuvanted with alum (1 μg + alum group), AS01_E_ (1 μg + AS01_E_ group) or AS03_B_ (1 μg + AS03_B_ group), and 4 μg/serotype/dose adjuvanted with alum (4 μg + alum group).

The formulations to be adjuvanted with AS01_E_ and AS03_B_ consisted of inactivated vaccine, vialed and freeze-dried. Each vial, corresponding to one dose, contained 1 μg of each DENV serotype. Dengue purified inactivated vaccine was reconstituted at the time of administration by mixing the freeze-dried product with the appropriate adjuvant system. To prepare DPIV with alum, monovalent bulk vaccine lots were combined to create the tetravalent formulation at either 1 μg/serotype/dose or 4 μg/serotype/dose. The formulated tetravalent bulk was adsorbed on alum for 1 hour and then vialed and stored at 2–8°C (36–46°F).

Phosphate-buffered saline was used as placebo. Placebo and vaccine injection volumes were identical (0.5 mL). The study was observer-blind, with vaccinations performed by study personnel not involved in the preparation of the vaccine formulations. Two doses of vaccine or placebo were given 4 weeks apart. All DPIV vaccine formulations and placebo were administered IM in the deltoid muscle at D0 and D28.

### Study participants.

Healthy male and female adults between 18 and 39 years of age who have lived in the Caribbean for more than 10 years were recruited at the University of Puerto Rico Medical Sciences Campus, Puerto Rico Clinical and Translational Research Consortium Center. Volunteers were provided with a detailed explanation of the study and enrolled after an informed consent process. Female participants had to be of nonchildbearing potential or abstinent, or had to use adequate contraceptive precautions for 30 days before vaccination, have a negative pregnancy test on the day of vaccination, and agreed to continue such precautions for 60 days after completion of the vaccination series. Volunteers seropositive for hepatitis B surface antigen, hepatitis C virus antibodies, or human immunodeficiency virus antibodies were excluded. Other exclusion criteria were a history of chronic disease; chronic alcohol consumption and/or drug abuse; and receipt of immunoglobulins and/or any blood products within 90 days preceding vaccination or had planned administration during the study period and laboratory test results outside normal limits for age, gender, and locality, at screening.

In total, 100 participants were planned to be enrolled and randomized 1:1:1:1:1 to receive one of the four DPIV formulations or saline placebo. The randomization was performed using MATEX, a program developed for use in SAS (Cary, NC).

### Safety evaluation.

The safety assessment was very similar to that described for our previous phase I study in the continental United States.^16^ Solicited injection site and general adverse events (AEs; grades 1–3) were recorded on diary cards for 7 days after each dose. Spontaneously reported AEs (coded with the use of the Medical Dictionary for Regulatory Activities^20^) were recorded for 28 days after each dose. Serious adverse events (SAEs), potential immune-mediated diseases (pIMDs), and medically attended events (MAEs) were recorded throughout the entire study period. Safety laboratory assessments were performed before 7 and 28 days after each vaccination, and 3 (M4), 6 (M7), 9 (M10), and 12 (M13) months after the second dose. All safety-related clinical laboratory values were reviewed and all abnormal values were assessed by the investigators as clinically significant or not, with respect to safety.

Enrollment of this trial occurred in two waves, starting with enrollment of 20 participants (four participants per group) and review of their safety data through 7 days postvaccination before enrollment of the remaining 80 participants. Three scheduled Safety Review Committee reviews of unblinded safety data took place: once 20 subjects had reached Day 7 post-Dose 1, once they reached Day 7 post-Dose 2, and once 60 subjects reached Day 7 postdose 1. Subjects have been followed for a total of 36 months post-Dose 2, in accordance with WHO recommendations for the clinical evaluation of dengue vaccines in endemic areas.^21^

### Immunogenicity assessment.

Blood samples were collected on the day of each vaccination and at D56, M4, M7, M10, and M13. To characterize DPIV vaccine humoral immunogenicity, anti-DENV–neutralizing antibodies (Nab) were measured before each dose and 28 days after (D28 and D56), and at M7 and M13. M4 and M10 were exploratory endpoints and assays were not available at the time of submission. Antibodies to each DENV type were measured at the Pilot Bioproduction Facility, WRAIR (Silver Spring, MD), using a 96-well quantitative microneutralization assay (MN50) performed in Vero cells as previously described.^9,12^ Seropositivity was defined as a titer ≥ 1:10. Antibody avidity, one measure of antibody quality, was determined for pre- and postvaccination sera as previously described.^16^ Peripheral blood mononuclear cells were collected for B and T cell assays. These assessments are ongoing and will be published at a later time.

### Statistical analysis.

This study was exploratory; thus, all analyses were descriptive, with no confirmatory statistical comparisons performed. The analysis of safety was performed on the total vaccinated cohort (TVC; i.e., all participants who received at least one vaccine dose), and the immunogenicity analysis was based on the according-to-protocol (ATP) cohorts for immunogenicity (participants who met all eligibility criteria, complied with protocol procedures, had no elimination criteria during the study, and had data available for at least one immunogenicity endpoint). Because 93% of all study subjects were seropositive for at least one dengue type before vaccination by MN50, the ATP analysis of immunogenicity was performed on primed subjects only.

The number and percentage of participants reporting each individual solicited local and general AE during the 7-day follow-up period was tabulated with exact 95% confidence intervals (CIs) after each vaccine dose and overall. The percentage of doses followed by each individual solicited local and general AE were tabulated with exact 95% CIs. All SAEs occurring during the study were listed for each treatment group. All SAEs, withdrawals due to AEs, pIMDs, pregnancies, and all related to treatment AEs were described in detail.

The percentage of participants with hematological and biochemical laboratory values within and outside (below or above) the normal ranges, and according to toxicity grading, were presented with exact 95% CIs at baseline and at each specified time point.

The immunogenicity parameters were calculated by group, with asymptotic 95% CIs for geometric mean titers (GMTs), and the geometric mean of fold increases in Nab from D0 for each DENV type. Participants with a titer below the assay cutoff were attributed the arbitrary value of half the cutoff for computation of GMT and fold increases. In addition, the geometric mean of fold increases between D56 and M13 was computed for each group and DENV serotype.

## RESULTS

### Study population.

**Figure 1.**
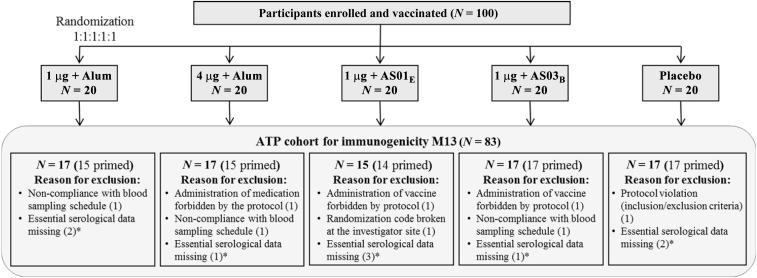
Disposition of study participants and reasons for exclusion from ATP cohort for immunogenicity. ATP = according-to-protocol; M13 = 12 months after dose 2; *N* = number of subjects in each group/cohort; * Withdrawals.

A total of 100 participants (20 per group) were enrolled and received two doses of vaccine or placebo (TVC); a total of 91 participants completed the study up to the M13 visit. Of the nine withdrawals, two participants permanently discontinued the study (one lost to follow-up and one moved to the continental United States) and seven migrated or moved from the study area without documenting a permanent discontinuation. Seventeen of the 100 study subjects (including the nine withdrawals) were excluded from the ATP cohort for immunogenicity M13 (Figure 1). The M13 ATP cohort for immunogenicity included 83 participants.

The mean age in the TVC at first vaccination was 27.9 years; 61% of participants were female (Table 1). All participants were American Hispanic or Latino.

**Table 1:** Demographic characteristics of the study participants (total vaccinated cohort)

Characteristics	1 μg + alum	4 μg + alum	1 μg + AS01_E_	1 μg + AS03_B_	Placebo	Total
	*N* = 20	*N* = 20	*N* = 20	*N* = 20	*N* = 20	*N* = 100
Age in years mean (SD)	26.9 (4.7)	27.7 (5.8)	27.9 (7.0)	28.9 (6.4)	28.3 (6.0)	27.9 (6.0)
Females, *n* (%)	13 (65%)	14 (70%)	9 (45%)	10 (50%)	15 (75%)	61 (61%)

1μg + alum indicates participants who received 1 μg/serotype/dose adjuvanted with alum; 4 μg + alum indicates participants who received 4 μg/serotype/dose adjuvanted with alum; 1 μg + AS01_E_ indicates participants who received 1 μg/serotype/dose adjuvanted with AS01_E_; 1 μg + AS03_B_ indicates participants who received 1 μg/serotype/dose adjuvanted with AS03_B_; *N* = number of participants; *n* (%) = number and percentage of participants in a specific category; SD = standard deviation.

### Reactogenicity and safety.

**Figure 2.**
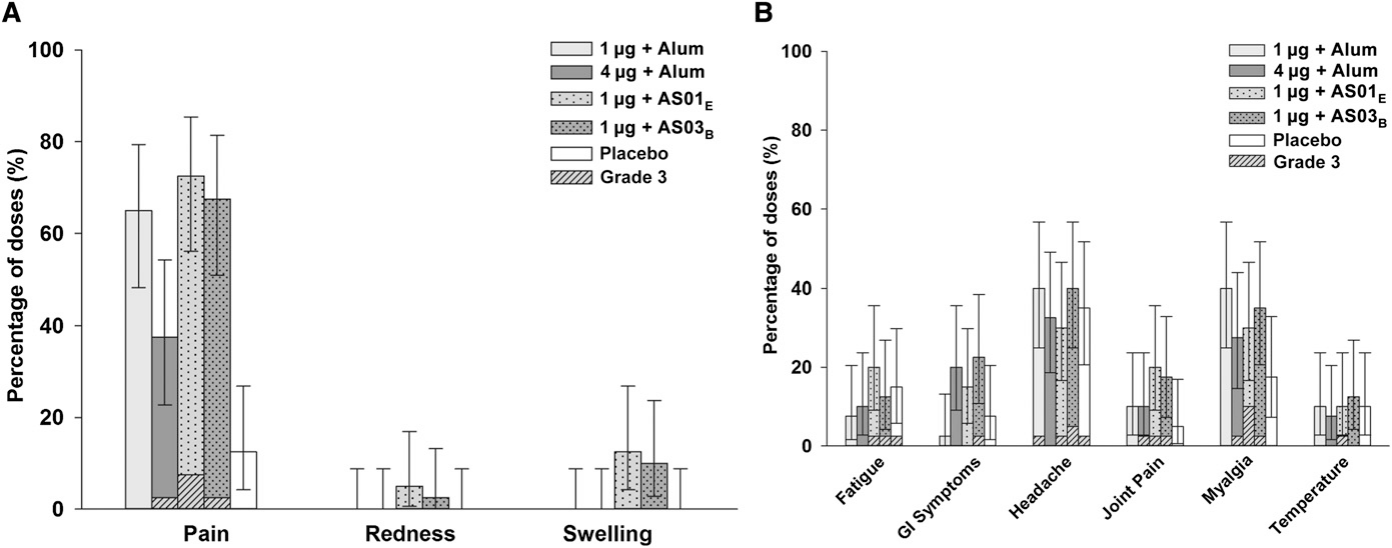
Overall per dose incidence of any grade solicited local (**A**) and general (**B**) adverse events during the 7-day postvaccination period (total vaccinated cohort). Error bars indicate exact 95% confidence intervals; GI = gastro-intestinal; Temperature = oral temperature ≥ 37.5°C (99.5°F).

All subjects returned a symptom diary card after vaccination. Most of the reported solicited injection site AEs were of mild or moderate intensity. Pain was more frequently reported in the vaccine groups than in the placebo group (Figure 2A). Grade 3 pain followed 1/40 (2.5%), 3/40 (7.5%), and 1/40 (2.5%) of doses in the 4 μg + alum, 1 μg +AS01_E,_ and 1 μg + AS03_B_ groups, respectively. No grade 3 swelling or redness was observed. Headache and myalgia of mild to moderate intensity were the most frequent solicited general AEs, reported with similar frequencies across all groups (Figure 2B). Grade 3 headache followed 1/40 (2.5%), 1/40 (2.5%), 2/40 (5%), and 1/40 (2.5%) of doses in the 1 μg + alum, 1 μg + AS01_E_, 1 μg + AS03_B_ and placebo groups, respectively. Grade 3 myalgia followed 1/40 (2.5%), 4/40 (10%), and 1/40 (2.5%) of doses in the 4 μg + alum, 1 μg + AS01_E,_ and 1 μg + AS03_B_ groups, respectively. Grade 3 fever followed 1/40 (2.5%) doses in the 1 μg + AS01_E_ group.

Concerning hematological and biochemical levels, there were four participants with a grade 3 or higher laboratory value: one participant in the placebo group had a grade 3 aspartate aminotransferase (AST) elevation at D28 (AST was 21 unit [U]/L at D0 [normal range: 15–37 U/L], 423 U/L at D28, and 27 U/L at D35. Alanine aminotransferase (ALT) was 171 U/L at D28 [normal range: 30–65 U/L] and AST and ALT returned to normal range within 7 days). Grade 3 anemia was reported by two participants in the 4 μg + alum group at M7 and M10 and by one participant in the 1 μg + AS03_B_ group at M7, M10, and M13. All grade 3 anemias were seen in women and were judged by the investigator to be likely due to iron deficiency (with varying degrees of compliance to oral therapeutic iron administration).

During the 28-day postvaccination period, the rates of subjects who reported at least one unsolicited AEs or at least one MAE ranged 80–90% and 10–20% across groups, respectively. There were 12 grade 3 unsolicited AEs judged by the investigator not related to vaccination: one upper respiratory tract infection and one dysmenorrhea in the 1 μg + alum group; one sinusitis, one hyperglycaemia, and one oropharyngeal pain in the 4 μg + alum group; one abdominal pain and one muscle spasms in the 1 μg + AS01_E_ group; one abdominal pain, one upper respiratory tract infection, one back pain, one dysmenorrhea, and one nasal congestion in the placebo group. No grade 3 vaccine-related unsolicited AEs were reported in the DPIV groups; one was reported in the placebo group (fatigue). The most frequently reported unsolicited AE was anemia that occurred in 40%, 60%, 35%, 45%, and 35% of the participants in the 1 μg + alum, 4 μg + alum, 1 μg + AS01_E_, 1 μg + AS03_B,_ and placebo groups, respectively.

Through M13 there were nine SAEs, all considered not related to vaccination, reported by two subjects in the 4 μg + alum group and two subjects in the placebo group. In the 4 μg + alum group, one subject reported bacterial gastroenteritis 355 days postdose 2, and the second subject reported spontaneous abortion approximately 5 weeks after last menstrual period, pyelonephritis, and hydronephrosis at 39, 309, and 310 days postdose 2, respectively. In the placebo group, one subject reported nephrolithiasis, urinary tract infection, and pyelonephritis at 27, 233, and 358 days postdose 2, and the second subject reported acute cholecystitis and cholelithiasis at 286 days postdose 2. Two pIMDs were reported: worsening of preexisting rheumatoid arthritis (not known to the study team at enrolment) in the 1 μg + AS03_B_ group and autoimmune thyroiditis at 334 days postdose 2 in the placebo group.

### Humoral immunogenicity.

**Figure 3.**
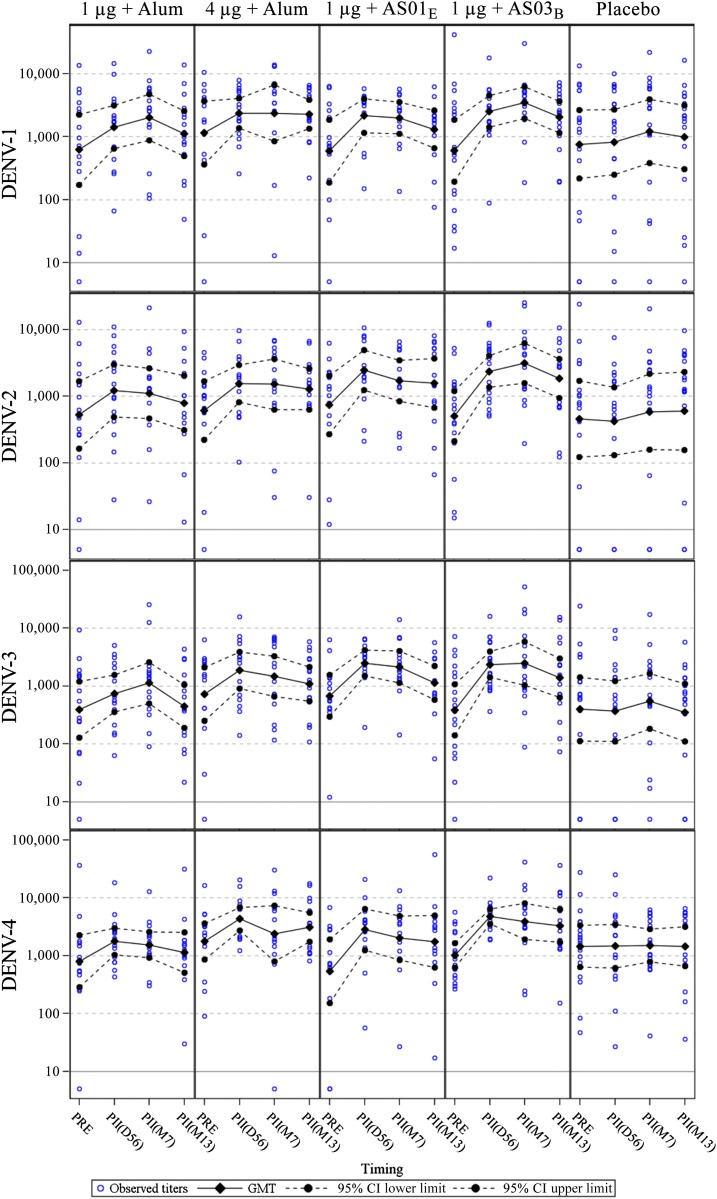
Geometric mean titers to DENV types 1–4 up to 1 year postdose 2 (according-to-protocol cohort for immunogenicity M13). Participants with a titer below the assay cutoff were attributed the arbitrary value of half the cutoff; D56 = Day 56 (1 month postdose 2); DENV = dengue virus type; M7 = month 7 (6 months postdose 2); M13 = month 13 (12 months postdose 2); PRE = prevaccination. This figure appears in color at www.ajtmh.org.

Of 100 study subjects enrolled, 93 were seropositive before vaccination for at least one dengue type by MN50. Eighty-two were tetravalent, three trivalent, three bivalent, and five monovalent, indicating that the vast majority of this young adult population had likely experienced more than one dengue infection. Seventy-eight of the 83 subjects included in the ATP cohort were seropositive for at least one dengue type (69 were tetravalent, three trivalent, two bivalent, and four monovalent). The ATP analysis of immunogenicity was performed on primed subjects only. Dengue virus Nab GMTs at D0, D56, M7, and M13 are presented by group in Figure 3 and Table 2. Neutralizing antibody titers for dengue seronegative subjects are shown in Supplemental Table 1. Geometric mean antibody titers prevaccination (D0) to all four DENV types were above 400 by group. Prevaccination Nab GMTs against DENV-1, DENV-2, and DENV-3 appeared comparable between treatment groups but DENV-4 prevaccination GMTs differed up to 3-fold between treatment groups.

**Table 2:** GMTs to each DENV serotype before vaccination and at D56, M7, and M13 after vaccination (ATP cohort for immunogenicity M13)

Serotype and group	Prevaccination (PRE)		Postdose 2 (D56)		Postdose 2 (M7)		Postdose 2 (M13)	
	*N*	GMT (95% CI)	*N*	GMT (95% CI)	*N*	GMT (95% CI)	*N*	GMT (95% CI)
DENV-1								
1 μg + alum	15	622 (172–2,247)	15	1,421 (641–3,149)	15	2,036 (875–4,740)	15	1,122 (494–2,548)
4 μg + alum	15	1,152 (361–3,677)	15	2,359 (1,378–4,038)	15	2,364 (846–6,600)	15	2,291 (1,352–3,883)
1 μg + AS01_E_	14	590 (187–1,861)	14	2,149 (1,162–3,971)	14	1,981 (1,109–3,539)	14	1,309 (662–2,590)
1 μg + AS03_B_	17	599 (195–1,845)	17	2,503 (1,406–4,458)	16	3,488 (1,966–6,191)	17	2,051 (1,156–3,641)
Placebo	17	758 (217–2,650)	17	822 (251–2,692)	17	1,223 (384–3,892)	17	983 (305–3,165)
DENV–2								
1 μg + alum	15	523 (163–1,679)	15	1,207 (489–2,982)	15	1,104 (467–2,611)	15	793 (312–2,017)
4 μg + alum	15	610 (221–1,687)	15	1,558 (820–2,960)	15	1,511 (627–3,641)	15	1,272 (628–2,576)
1 μg + AS01_E_	14	741 (271–2,028)	14	2,451 (1,230–4,887)	14	1,714 (846–3,470)	14	1,567 (668–3,675)
1 μg + AS03_B_	17	500 (210–1,191)	17	2,355 (1,357–4,086)	16	3,148 (1,578–6,281)	17	1,847 (945–3,609)
Placebo	17	457 (123–1,699)	17	421 (131–1,356)	17	585 (158–2,169)	17	603 (157–2,316)
DENV–3								
1 μg + alum	15	390 (128–1,192)	15	745 (356–1,561)	15	1,140 (505–2,573)	15	447 (191–1,050)
4 μg + alum	15	722 (249–2,090)	15	1,870 (903–3,871)	15	1,482 (664–3,307)	15	1,092 (551–2,162)
1 μg + AS01_E_	14	681 (297–1,563)	14	2,497 (1,497–4,163)	14	2,149 (1,145–4,033)	14	1,139 (580–2,240)
1 μg + AS03_B_	17	387 (140–1,073)	17	2,373 (1,423–3,958)	16	2,470 (1,037–5,884)	17	1,389 (641–3,011)
Placebo	17	401 (113–1,417)	17	369 (110–1,235)	17	550 (183–1,652)	17	349 (110–1,099)
DENV–4								
1 μg + alum	15	804 (285–2,273)	15	1,770 (1,045–2,997)	15	1,544 (927–2,570)	15	1,143 (515–2,535)
4 μg + alum	15	1,766 (856–3,646)	15	4,332 (2,755–6,812)	15	2,408 (797–7,276)	15	3,133 (1,756–5,593)
1 μg + AS01_E_	14	539 (152–1,912)	14	2,842 (1,242–6,503)	14	2,028 (851–4,834)	14	1,751 (622–4,929)
1 μg + AS03_B_	17	1,015 (622–1,657)	17	4,787 (3,551–6,452)	16	3,930 (1,904–8,113)	17	3,268 (1,703–6,268)
Placebo	17	1,461 (637–3,352)	17	1,467 (620–3,471)	17	1,511 (783–2,916)	17	1,441 (659–3,148)

1μg + alum indicates participants who received 1 μg/serotype/dose adjuvanted with alum; 4 μg + alum indicates participants who received 4 μg/serotype/dose adjuvanted with alum; 1 μg + AS01_E_ indicates participants who received 1 μg/serotype/dose adjuvanted with AS01_E_; 1 μg + AS03_B_ indicates participants who received 1 μg/serotype/dose adjuvanted with AS03_B_; ATP = according-to-protocol; DENV = dengue virus; GMTs = geometric mean antibody titers calculated on all subjects; *N* = number of subjects with available data; Postdose 2 (D56) = blood sampling 28 days postdose 2 at Day 56; Post-dose 2 (M7) = blood sampling 6 months post-dose 2; Postdose 2 (M13) = blood sampling 12 months postdose 2; Prevaccination (PRE) = blood sampling prevaccination at Day 0; 95% CI = 95% confidence interval.

By D56, GMTs for each DENV type had increased approximately by 2-fold in the alum-adjuvanted groups and approximately by 3- to 6-fold in the AS01_E_ and AS03_B_ groups (Table 3A). In all vaccine groups, GMTs peaked either at D56 or M7 and waned modestly through M13 (Figure 3), but remained well above prevaccination titers (Table 2). The overall ranking of the observed M13/PRE GMT ratios was 1 μg + AS03_B_ (3.22–3.70 range) > 1 μg + AS01_E_ (1.67–3.25) > 4 μg + alum (1.51–2.09) > 1 μg + alum (1.15–1.80) (Table 3B). M13/D56 ratios, as an estimate of waning kinetics, ranged between 0.46 and 0.97 (Table 3C).

**Table 3:** Ratios between GMTs to each DENV serotype at 28 days (D56) after the second vaccine dose and before vaccination (PRE) (A); between 12 months after the second vaccine dose (M13) and PRE (B); and between M13 and D56 (C) (ATP cohort for immunogenicity M13)

	DENV-1	DENV-2	DENV-3	DENV-4
A. D56/PRE GMTs				
1 μg + alum (*N* = 15)	2.29	2.31	1.91	2.20
4 μg + alum (*N* = 15)	2.05	2.55	2.59	2.45
1 μg + AS01_E_ (*N* = 14)	3.64	3.31	3.66	5.28
1 μg + AS03_B_ (*N* = 17)	4.18	4.71	6.13	4.72
Placebo (*N* = 17)	1.08	0.92	0.92	1.00
B. M13/PRE GMTs				
1 μg + alum (*N* = 15)	1.80	1.52	1.15	1.42
4 μg + alum (*N* = 15)	1.99	2.09	1.51	1.77
1 μg + AS01_E_ (*N* = 14)	2.22	2.11	1.67	3.25
1 μg + AS03_B_ (*N* = 17)	3.42	3.70	3.59	3.22
Placebo (*N* = 17)	1.30	1.32	0.87	0.99
C. M13/D56 GMTs				
1 μg + alum (*N* = 15)	0.79	0.66	0.60	0.65
4 μg + alum (*N* = 15)	0.97	0.82	0.58	0.72
1 μg + AS01_E_ (*N* = 14)	0.61	0.64	0.46	0.62
1 μg + AS03_B_ (*N* = 17)	0.82	0.78	0.59	0.68
Placebo (*N* = 17)	1.20	1.43	0.94	0.98

1μg + alum indicates participants who received 1 μg/serotype/dose adjuvanted with alum; 4 μg + alum indicates participants who received 4 μg/serotype/dose adjuvanted with alum; 1 μg + AS01_E_ indicates participants who received 1 μg/serotype/dose adjuvanted with AS01_E_; 1 μg + AS03_B_ indicates participants who received 1 μg/serotype/dose adjuvanted with AS03_B_; ATP = according-to-protocol; DENV = dengue virus; GMTs = geometric mean antibody titers calculated on all subjects; Postdose 2 (D56) = blood sampling 28 days postdose 2 at Day 56; Postdose 2 (M13) = blood sampling 12 months postdose 2; Prevaccination (PRE) = blood sampling prevaccination at Day 0.

In addition to Nab responses, antibody avidity was determined pre- and postvaccination. The vast majority of study subjects entered the study with high-avidity dengue-specific antibody indices. After vaccination, an increase in avidity was observed for some of the subjects who entered the study with relatively low avidity for a given dengue serotype. The AS03 treatment group seemed most consistent in this regard (Supplemental Figures 1–4). Across all treatment groups, high Nab titers after dose 1 correlated with high-avidity indices (Supplemental Figure 5).

## DISCUSSION

Results from this first phase I study of a new vaccine candidate with inactivated DENV in a dengue- primed population showed that all four DPIV vaccine formulations were well tolerated and immunogenic. Transient mild to moderate injection site pain was more frequently reported in active treatment groups, but all other solicited local and general solicited AEs were balanced between active and control groups. Although one could have speculated that more reactogenicity will be observed in a young dengue-primed population, reactogenicity in this study population was similar to that observed in a similar trial in naive subjects (NCT01666652).^16^ Of note, only one grade 3 fever (duration of 1 day) was reported and elevated temperatures were uncommon and balanced between active treatment groups and the placebo group. Anemia was the most frequently observed laboratory abnormality in active and control groups and was more frequently observed in women than in men. The prevalence of anemia observed in this study was comparable to that observed in the general Puerto Rican population, and was judged independently by the medical monitor not related to the study vaccine. No SAEs related to vaccination were reported in any group through the M13 visit.

A number of investigational vaccines have been evaluated for their ability to boost dengue Nab titers in dengue-primed subjects and subjects primed via exposure to one or more wild-type dengue infections are expected to respond to vaccination with a rise to all four dengue types.^22^ Our findings are in line with these expectations: in all DPIV groups, Nab GMTs against all four DENV types rose after vaccination, waned only modestly (generally less than 2-fold), and stayed above baseline for at least 1 year. The fold rise of Nab titers depended on antigen dose and on the adjuvant used. Similar to what was observed for other antigens, for example, influenza, malaria, or herpes zoster virus,^23–26^ a more potent response and a dose-sparing effect were observed when using complex adjuvant systems such as AS01_E_ and AS03_B_ rather than alum.

Studies conducted by Dorigatti et al.^27^ suggest that after vaccination with an LAV (chimeric yellow fever/dengue-tetravalent dengue vaccine [first dengue vaccine to be marketed under the trade name “Dengvaxia”]), Nab titers increased less in populations with high Nab titers prevaccination, and one could hypothesize that the observed reduced fold rise was either due to restriction of vaccine virus infectivity or replication, or due to a saturation effect, that is, that titers cannot increase above a certain upper limit. In our study, we did not observe a saturation effect in a multitypic positive population with high titers prevaccination. The GMT fold rise increased with antigen dose and when more potent adjuvants were used. In multitypic positive subjects receiving AS03_B_-adjuvanted vaccine, GMTs rose between 4- and 6-fold, depending on the DENV type. Dorigatti et al.^27^ reported approximately 2-fold rises. Dengue virus–specific antibody-avidity indices were already high prevaccination in this highly dengue-experienced population, and an additional rise in avidity was only observed for several subjects who entered the study with relatively low indices. Across all treatment groups, high Nab titers correlated with high-avidity indices, likely as a result of both prior dengue exposure and vaccination.

Whether DPIV induces or recalls homotypic antibody responses against all four DENV serotypes is difficult to assess in this dengue-primed population. In a similar study conducted in mostly naive subjects,^16^ high titer balanced responses to all four DENV types were observed after two doses of vaccine administered 1 month apart, and the ranking of GMTs by adjuvant and antigen dose was the same as observed here. The data in dengue-naive subjects speak against immunodominance of a single serotype after vaccination with DPIV, in contrast to observations reported for two LAVs in development.^27,28^ Antibody depletion studies have shown that after primary dengue infection, type-specific antibodies represent only a small subset of the repertoire of dengue-reactive antibodies but are crucial for neutralization activity.^29^ However, after secondary infection with wild-type viruses or monovalent vaccine viruses, complex-reactive (pan DENV) and group-reactive (pan-flavivirus) antibodies seem to contribute significantly to neutralization activity against nonexposed serotypes.^22^ For this present study, we have not yet performed depletion studies and, thus, no conclusions can be drawn regarding type and complex specificity.

Although Nab GMTs waned considerably in flavivirus-naive population from D56 to M7,^16^ very little waning was observed in this study. This finding, although expected, indicates that the analysis of immune responses—and of efficacy endpoints in phase 3 pivotal trials—should probably be carried out separately for dengue-primed and dengue-naive populations. Waning of Nab titers and the efficacy and safety implications of the same also supports the importance of assessing postvaccination Nab kinetics over a period of time remote from vaccination to better understand kinetics and Nab final late plateau titers. The results from phase II and phase III dengue vaccine studies conducted with CYD-TDV suggest that immune response and efficacy differ based on serostatus, and Dorigatti et al.^27^ conclude that once serostatus was accounted for, factors such as age (past infancy) does not play a significant role in a subject’s immune response.^30^

Although Nab GMTs in our study are higher than those previously reported after LAV formulations in flavivirus-primed adults in Thailand measured with the same MN50 assay,^13^ a direct comparison of titers between live and nonreplicating vaccines is prone to misinterpretation. Live-attenuated vaccines expressing all dengue nonstructural proteins may induce a broader spectrum and intensity of responses, making a comparison limited to the quantitation of in vitro neutralization superficial. For instance, we still need to evaluate the cell mediated responses induced by this candidate vaccine, and will do so both in dengue-naive and primed subjects. Cellular immune responses analyses are still ongoing and will be reported in a separate publication.

The study reported here has several limitations. First and foremost, this is a small trial with only 20 subjects per group. The study was not powered to detect differences between formulations and all analyses are descriptive only. Secondly, the vaccinated population was > 90% tetravalent positive at baseline. This did not allow for a comparison between monovalent positive and multivalent positive subjects. Lastly, a number of subjects moved from Puerto Rico to the United States mainland and could not be followed through M13 for immunogenicity.

## CONCLUSION

This new investigational DPIV vaccine had an acceptable safety profile in a small number of flavivirus-primed healthy adult subjects and all formulations boosted Nab responses, with complex adjuvants increasing immunogenicity versus alum adjuvantation. Neutralizing antibody titers remained high (and above baseline titers) through M13. These results encourage continuation of DPIV clinical development.

**Figure 4.**
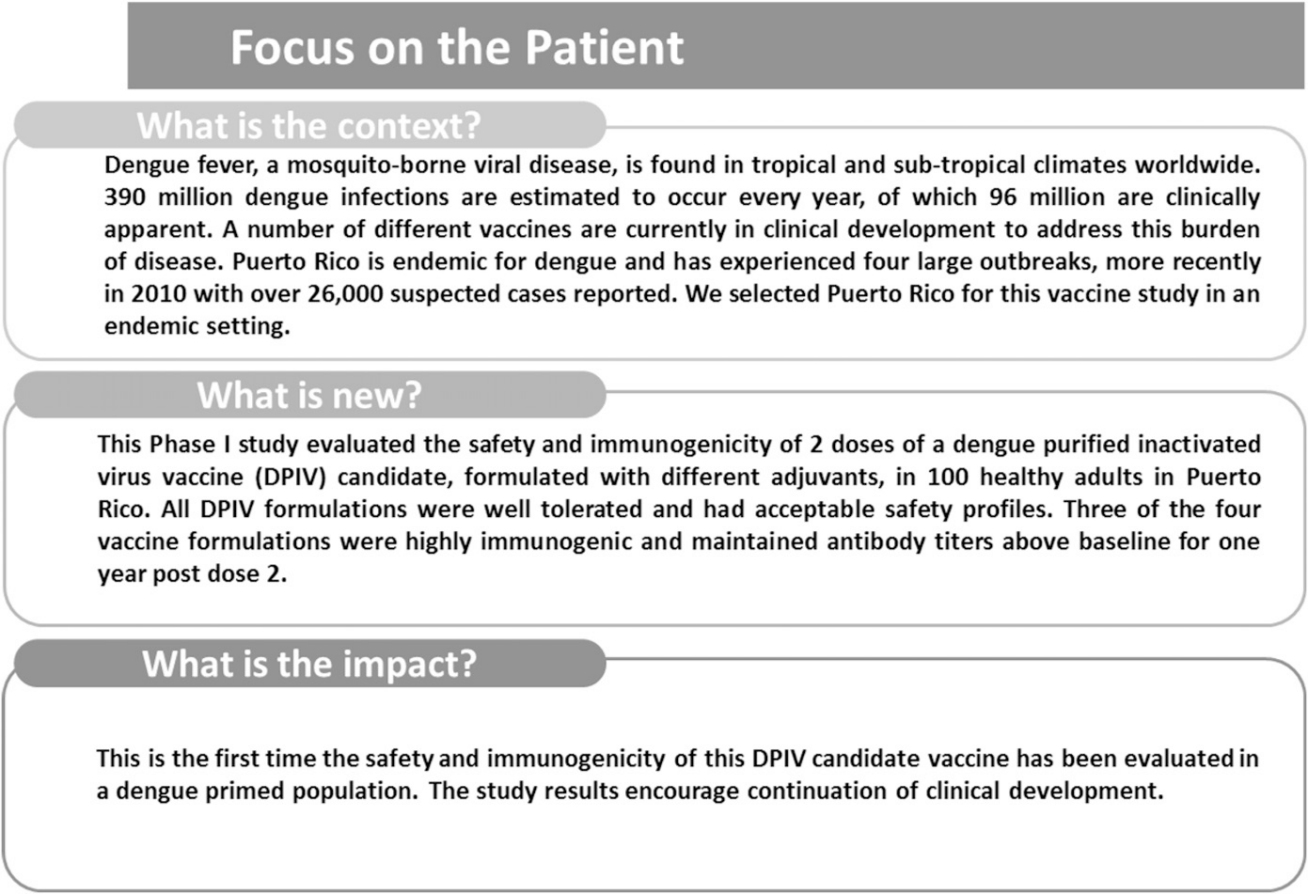
Focus on patient section.

A graphical summary contextualizing the results and potential clinical research relevance and impact is displayed in the Focus on Patient Section (Figure 4) for the benefit of health-care professionals.

## Supplementary Material

Supplemental Table and Figure.
