# Supplementary material for: Phase I Randomized Study of a Tetravalent Dengue Purified Inactivated Vaccine in Healthy Adults from Puerto Rico
Source: Am J Trop Med Hyg. 2018 Mar 5;98(5):1435–43. doi: 10.4269/ajtmh.17-0627 (PMC5953365; doi:10.4269/ajtmh.17-0627)
Supplement: Supplementary file 1 [file tpmd170627.SD1.pdf]

Supplementary Table 1

Neutralizing antibody titers for unprimed subjects (ATP cohort for immunogenicity M13)

| Pid | Group | Timing | MN50 | | | |
|---|---|---|---|---|---|---|
| | | | DENV-1 | DENV-2 | DENV-3 | DENV-4 |
| 15 | 4 µg + alum | PRE | < 10 | < 10 | < 10 | < 10 |
| | | PII(D56) | 74 | 58 | 70 | 114 |
| | | PII(M7) | 14 | < 10 | < 10 | < 10 |
| | | PII(M13) | < 10 | < 10 | < 10 | < 10 |
| 20 | 1 µg + alum | PRE | < 10 | < 10 | < 10 | < 10 |
| | | PII(D56) | 80 | 20 | 56 | 20 |
| | | PII(M7) | < 10 | < 10 | < 10 | < 10 |
| | | PII(M13) | < 10 | < 10 | < 10 | < 10 |
| 85 | 1 µg + $AS01_E$ | PRE | < 10 | < 10 | < 10 | < 10 |
| | | PII(D56) | 155 | 335 | 1,070 | 142 |
| | | PII(M7) | 2,116 | 1,801 | 3,422 | 3,715 |
| | | PII(M13) | 13 | < 10 | 22 | < 10 |
| 101 | 1 µg + alum | PRE | < 10 | < 10 | < 10 | < 10 |
| | | PII(D56) | 56 | 70 | 61 | 69 |
| | | PII(M7) | < 10 | 24 | 25 | < 10 |
| | | PII(M13) | < 10 | 17 | < 10 | < 10 |
| 146 | 4 µg + alum | PRE | < 10 | < 10 | < 10 | < 10 |
| | | PII(M7) | < 10 | < 10 | < 10 | < 10 |
| | | PII(M13) | < 10 | < 10 | < 10 | < 10 |

Unprimed, participants with negative titer for all the dengue virus (DENV) serotype at baseline; 1 µg + alum indicates participants who received 1 µg/serotype/dose adjuvanted with alum; 4 µg + alum indicates participants who received 4 µg/serotype/dose adjuvanted with alum; 1 µg + $AS01_E$ indicates participants who received 1 µg/serotype/dose adjuvanted with AS01E; ATP = according-toprotocol; MN50 = 96-well quantitative microneutralization assay; PRE = blood sampling prevaccination at Day 0; PII(D56) = blood sampling 28 days postdose 2 at Day 56; PII(M7) = blood sampling 6 months postdose 2; PII(M13) = blood sampling 12 months postdose 2.

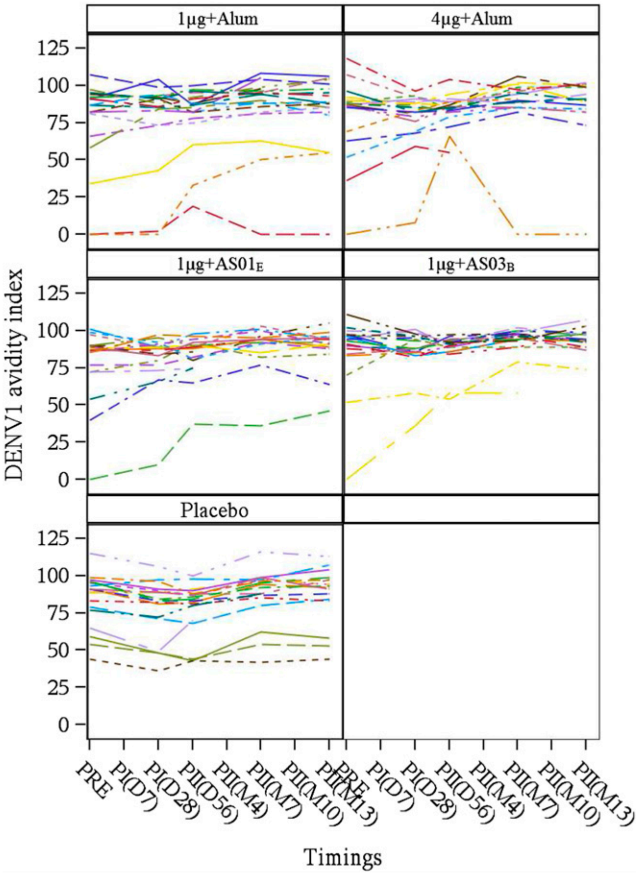


Supplemental Figure 1. Kinetic profile for DENV-1 avidity index for the subjects having received dose 2 (adapted ATP cohort for immunogenicity). µg + alum indicates participants who received 1 µg/serotype/dose adjuvanted with alum; 4 µg + alum indicates participants who received 4 µg/serotype/dose adjuvanted with alum; 1 µg + $AS01_E$ indicates participants who received 1 µg/serotype/dose adjuvanted with $AS01_E$; 1 µg + $AS03_B$ indicates participants who received 1 µg/serotype/dose adjuvanted with $AS03_B$; ATP = according-to-protocol; DENV = dengue virus; D7 and D28 = postdose 1 visit at Day 7 and 28, respectively; D56 = blood sampling 28 days postdose 2 at Day 56; M7 = blood sampling 6 months postdose 2; M13 = blood sampling 12 months postdose 2; PRE = blood sampling prevaccination at Day 0.

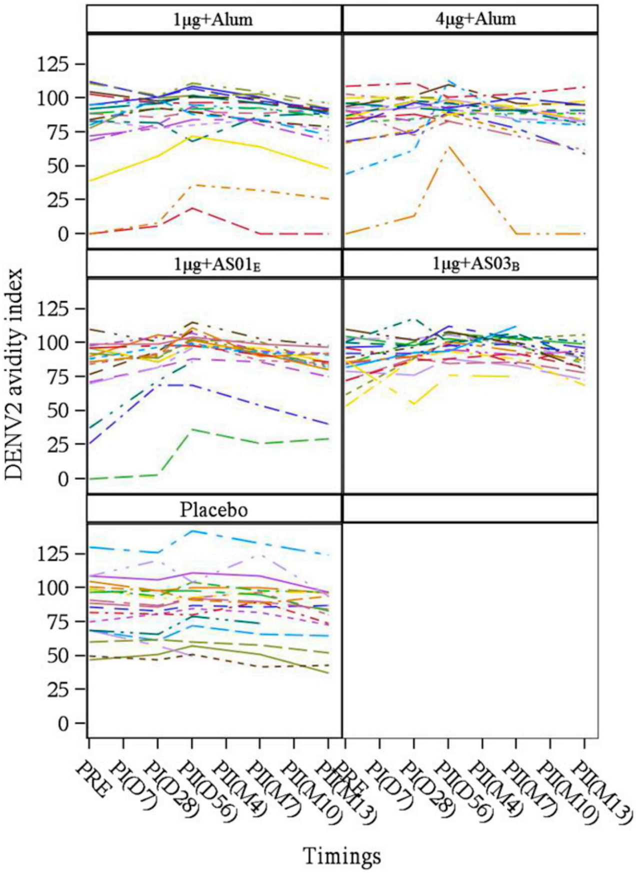


Supplemental Figure 2. Kinetic profile for DENV-2 avidity index for the subjects having received dose 2 (adapted ATP cohort for immunogenicity). 1 µg + alum indicates participants who received 1 µg/serotype/dose adjuvanted with alum; 4 µg + alum indicates participants who received 4 µg/serotype/dose adjuvanted with alum; 1 µg + $AS01_E$ indicates participants who received 1 µg/serotype/dose adjuvanted with $AS01_E$; 1 µg + $AS03_B$ indicates participants who received 1 µg/serotype/dose adjuvanted with $AS03_B$; ATP = according-to-protocol; DENV = dengue virus; D7 and D28 = post-dose 1 visit at Day 7 and 28, respectively; D56 = blood sampling 28 days postdose 2 at Day 56; M7 = blood sampling 6 months postdose 2; M13 = blood sampling 12 months postdose 2; PRE = blood sampling prevaccination at Day 0.

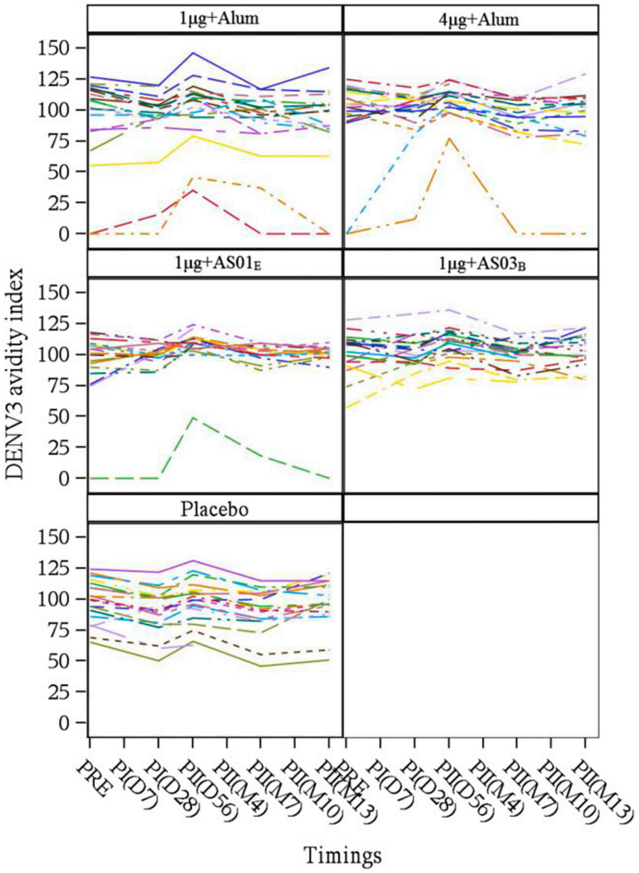


Supplemental Figure 3. Kinetic profile for DENV-3 avidity index for the subjects having received dose 2 (adapted ATP cohort for immunogenicity). µg + alum indicates participants who received 1 µg/serotype/dose adjuvanted with alum; 4 µg + alum indicates participants who received 4 µg/serotype/dose adjuvanted with alum; 1 µg + $AS01_E$ indicates participants who received 1 µg/serotype/dose adjuvanted with $AS01_E$; 1 µg + $AS03_B$ indicates participants who received 1 µg/serotype/dose adjuvanted with $AS03_B$; ATP = according-to-protocol; DENV = dengue virus; D7 and D28 = postdose 1 visit at Day 7 and 28, respectively; D56 = blood sampling 28 days postdose 2 at Day 56; M7 = blood sampling 6 months postdose 2; M13 = blood sampling 12 months postdose 2; PRE = blood sampling prevaccination at Day 0.

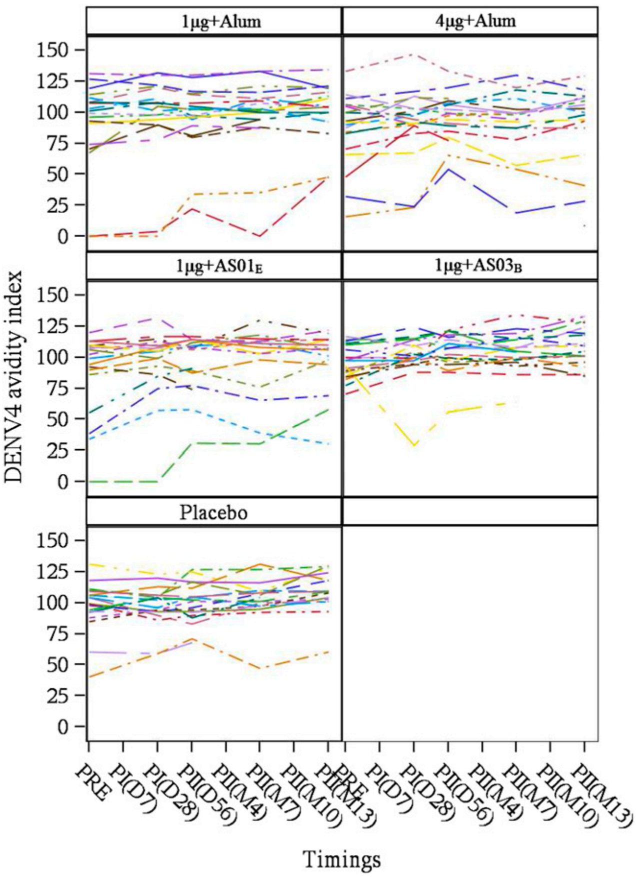


Supplemental Figure 4. Kinetic profile for DENV-4 avidity index for the subjects having received dose 2 (adapted ATP cohort for immunogenicity). 1 μg + alum indicates participants who received 1 μg/serotype/dose adjuvanted with alum; 4 μg + alum indicates participants who received 4 μg/serotype/dose adjuvanted with alum; 1 μg + $AS01_E$ indicates participants who received 1 μg/serotype/dose adjuvanted with $AS01_E$; 1 μg + $AS03_B$ indicates participants who received 1 μg/serotype/dose adjuvanted with $AS03_B$; ATP = according-to-protocol; DENV = dengue virus; D7 and D28 = postdose 1 visit at Day 7 and 28, respectively; D56 = blood sampling 28 days post-dose 2 at Day 56; M7 = blood sampling 6 months postdose 2; M13 = blood sampling 12 months post-dose 2; PRE = blood sampling prevaccination at Day 0.

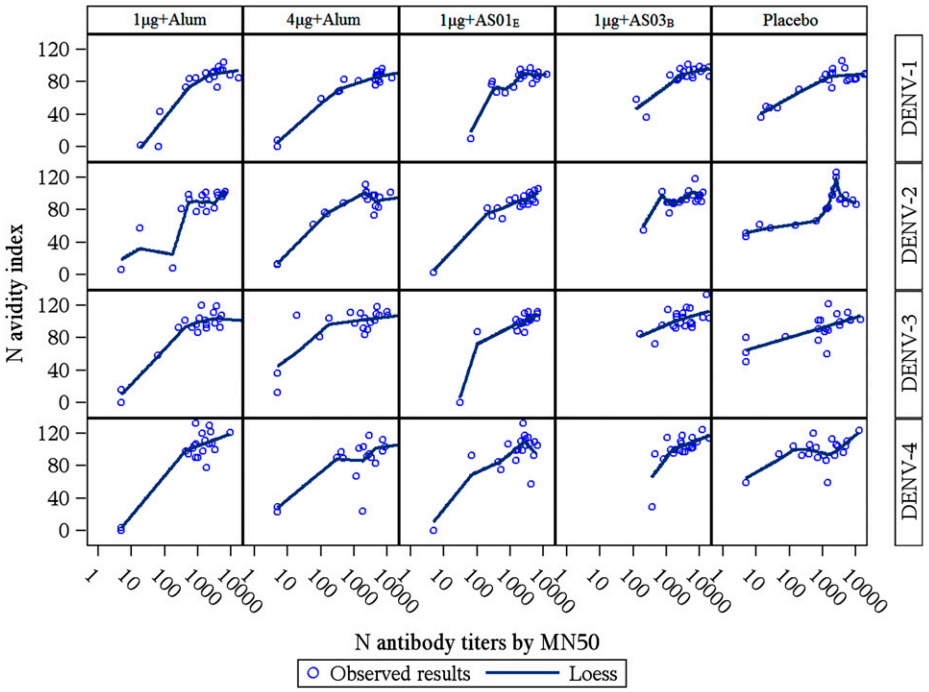


Supplemental Figure 5. *N* avidity index (natural scale) at Day 28 visit in function of *N* antibody titers by MN50 method (log-scale) at day 28 visit by group and for each dengue serotype (total vaccinated cohort). 1 μg + alum indicates participants who received 1 μg/serotype/dose adjuvanted with alum; 4 μg + alum indicates participants who received 4 μg/serotype/dose adjuvanted with alum; 1 μg + $AS01_E$ indicates participants who received 1 g/serotype/dose adjuvanted with $AS01_E$; 1 μg + $AS03_B$ indicates participants who received 1 μg/serotype/dose adjuvanted with $AS03_B$; DENV = dengue virus; Placebo = saline control. Note: all subjects with available results in both assays were included and negative antibody titers were given the arbitrary value of half the cutoff (5).
